# Single-Layer Wide-Angle Scanning Linear Phased Arrays Based on Multimode Microstrip Patch Elements

**DOI:** 10.3390/mi15010003

**Published:** 2023-12-19

**Authors:** Dongsheng Li, Jie Yang, Jianing Zhao, Yongzhen Dong, Hao Li, Tianming Li, Haiyang Wang, Biao Hu, Yihong Zhou, Fang Li, Ruoyang Yang

**Affiliations:** 1 The 54th Research Institute of China Electronics Group Corporation, Shijiazhuang 050081, China; 15833967185@163.com; 2 Beijing Research Institute of Telemetry, Beijing 100076, China; 3 College of Computer Science and Engineering, Guilin University of Technology, Guilin 541006, China; dong1029yong@163.com (Y.D.); lexi.f.li@outlook.com (F.L.); yry935@163.com (R.Y.); 4 Guangxi Key Laboratory of Embedded Technology and Intelligent System, Guilin University of Technology, Guilin 541006, China; 5 Yangtze Delta Region Institute (Huzhou), University of Electronic Science and Technology of China, Huzhou 313001, China; tianming@uestc.edu.cn (T.L.); hhywa@uestc.edu.cn (H.W.); hubiao2112@126.com (B.H.); zhouyh@uestc.edu.cn (Y.Z.); 6 School of Electronic Science and Engineering, University of Electronic Science and Technology of China, Chengdu 610054, China

**Keywords:** phased array, wide beamwidth, wide-angle scanning, multimode

## Abstract

This paper introduces a novel single-layer microstrip patch element designed to achieve a wide beamwidth, in order to address the growing demand for wide-angle scanning capabilities in modern phased array systems. The proposed element, comprising a slot-etched circular patch and an array of metallized holes arranged in square rings, offers a unique approach to beam shaping. By carefully adjusting parameters such as the slot structure and feeding position, our element is engineered to simultaneously excite both the TM_01_ and TM_21_ modes, a key feature that contributes to its wide beamwidth characteristics. Through the constructive interference of these modes, our element demonstrates a remarkable 3 dB beamwidth of approximately 150° in both principal planes, showcasing its potential for wide-angle scanning applications. To validate the practical performance of this proposed element, two linear phased arrays are manufactured and experimentally evaluated. The simulation results confirm the wide-angle scanning capability of the antennas in both the E-plane and H-plane. Furthermore, the experimental assessment demonstrates that these linear phased arrays can effectively generate scanning beams within a frequency range of 25 GHz to 28 GHz, covering a wide angular range from −60° to 60°, while maintaining a gain loss within 3 dB. This innovative design approach not only offers a promising solution for achieving a wide beamwidth in microstrip patch elements, but also holds significant potential for the development of cost-effective phased arrays with wide-angle scanning capabilities, making it a valuable contribution to the advancement of phased array technology.

## 1. Introduction

Phased array antennas, a type of advanced antenna system, achieve beam scanning and pattern shaping by dynamically adjusting the phase of individual radiating elements [1]. This phase manipulation allows for the electronic control of the antenna’s main lobe direction, enabling the formation of multiple beams with high flexibility and accuracy. The ability to alter the antenna pattern’s maximum value direction through phase control contributes to the adaptability and versatility of phased arrays, making them a subject of great interest to researchers. Furthermore, the potential applications of phased arrays in military radar and civilian communication systems are significant, given their capability to support multiple channels and their suitability for various communication and sensing tasks. However, a major limitation of a typical phased array is that its main beam can only scan within a range of approximately −50° to 50°, resulting in a loss of gain of around 4–5 dB [2,3,4,5,6]. In an effort to expand the scope of scanning, preserve the uniformity of scanning gain, and improve the performance of wide-angle scanning, numerous endeavors have been undertaken throughout the literature. Initially, the prevalent method involved employing mechanical rotation in conjunction with phased shifting. In [7], a strategy employing a multi-panel configuration for phased arrays was introduced to achieve wide-angle scanning capabilities. By altering the orientation of each element, this array is capable of scanning its beam within a range of approximately −70° to 70°. Subsequently, pattern reconfigurable elements were incorporated into the development of wide-angle phased arrays, enabling the modification of the antenna radiation pattern via current distribution adjustments, while maintaining other antenna characteristics [8,9,10,11,12,13]. In [8], the proposed design involves a microstrip Yagi antenna element that incorporates switchable parasitic strips. This feature allows for the manipulation or adjustment of the radiation pattern, thereby facilitating the scanning of the main beam within a range of −60° to 60°. Additionally, this design ensures that the gain fluctuation remains below 3 dB.

In recent years, the research emphasis on wide scanning angle phased arrays has primarily centered on wide-beamwidth elements [14,15,16,17,18,19,20,21]. These arrays eliminate the need for extra circuits and components found in previous pattern reconfigurable elements, streamlining array design while retaining crucial scanning attributes. Diverse techniques exist for broadening the beamwidth of phased array elements, including novel microstrip magnetic dipole antenna structures [14], parasitic pixel layers [15], and the utilization of electric walls [16,17]. However, it is common for these works to exhibit a wide beamwidth solely in either the E-plane or H-plane. Therefore, it is difficult for phased array to achieve a wide scanning range in both planes. One such example is the meander line element proposed by K S. Beenamole et al. [21] in the S-band. While their element demonstrates symmetrical radiation patterns in the E-plane with a beamwidth exceeding 130°, the beamwidth in the H-plane is approximately 80°, consequently constraining the scanning range of the ultimate phased array in the H-plane as well. The patch mode theory presents a promising approach to address the aforementioned issues, as it enables the generation of wide-beamwidth radiation or the expansion of the impedance bandwidth by exciting multiple modes [22,23,24,25,26,27]. This technique has been successfully employed in the development of wide scanning angle phased arrays [22]. In the present study, a groundbreaking multimode patch element is proposed, capable of selectively exciting the TM_10_/TM_01_ mode of a slot-etched square patch and the TM_21_ mode of a circular patch. By strategically switching and combining these excitation modes, the generation of wide-beamwidth radiation with consistent polarization is achieved in two principal planes. A 64-element phased array, utilizing this specific element, has been designed to achieve dual-polarized wide-angle scanning capabilities in both planes. In [24], a novel design approach for a dual-mode, wideband circular sector patch antenna based on an approximate 1.5-wavelength magnetic dipole model and cavity model is proposed. It introduces design criteria, theoretical analysis, and experimental validations for the antenna’s operational modes, radiation behaviors, and impedance bandwidth enhancement. The proposed design approach is experimentally verified in air and modified Teflon substrates. The fabricated antenna demonstrates stable, high boresight gain and dual-resonant characteristics, validating the effectiveness of this design approach. Additionally, it also discusses the potential for applications in array antennas without grating lobes and addresses the challenge of designing a wideband patch antenna with a stable unidirectional radiation and a reduced size, sidelobe level, and complexity.

To sum up, a multimode patch element based on a single-layer substrate is proposed in this paper. A wide beamwidth can be achieved by simultaneously exciting the TM_01_ mode and TM_21_ mode on a slot-etched circular patch through the adjustment of certain parameters and the feeding position. In order to reduce the mutual coupling among the elements within the arrays, a certain number of metallized holes are strategically positioned surrounding the slot-etched circular patch. The simulation results based on optimized parameters demonstrate that the proposed element exhibits a beamwidth in proximity to 160° on both main planes. Based on this element, two linear phased arrays are designed, fabricated, and measured. The simulation results and experimental results collectively indicate that these two linear phased arrays have a scanning capability of ±60° within a frequency range spanning from 25 GHz to 28 GHz, and that their gain fluctuation is less than 3 dB.

## 2. Proposed Wide-Beamwidth Element

**Figure 1:**
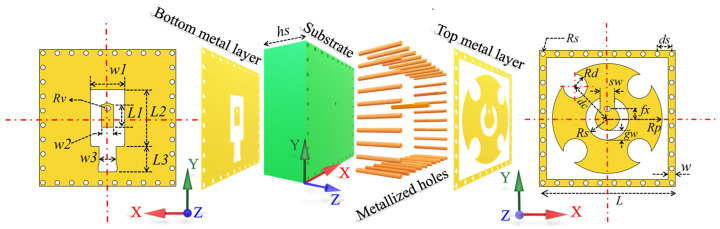
Geometry schematic for the proposed wide-beamwidth element.

The geometry of the multimode patch element is depicted in Figure 1. The element is composed of two metal layers and a single-layer substrate. On the top metal layer, the main part is a circular patch and a square ring patch. The radius of the circular patch is *Rp*. The side length and width of the square ring patch are *L* and *w*, respectively. In order to achieve multimode resonance, the circular patch is modified by subtracting four small circular patches, positioned diagonally, and a concentric split-ring shape. The radius and width of the split ring are denoted as *Rs* and *gw*, respectively, and the opening size of the split ring is *sw*. The radius of all four small circular patches is *Rd*, and the distance between the center of these circular patches and the center of the element is expressed as *dc*. In order to reduce the mutual coupling between elements, metallized holes are arranged in the square ring patch [28]. The radius and spacing of the metallized holes are *Rm* and *ds*, respectively. A substrate possessing a relative permittivity and a loss angle of 0.003 is positioned beneath the slot-etched circular patch and square ring patch to provide support. The thickness of the substrate is *hs*. A distinctive shape is etched onto the bottom layer primarily to prevent the occurrence of short circuits during the welding process with the SSMP connector. Additionally, the top and bottom layers of the element are interconnected via a metallized hole with an offset of *fx*; the radius of this metallized hole is denoted as *Rv*. More detailed parameters can be found in Figure 1.

**Figure 2:**
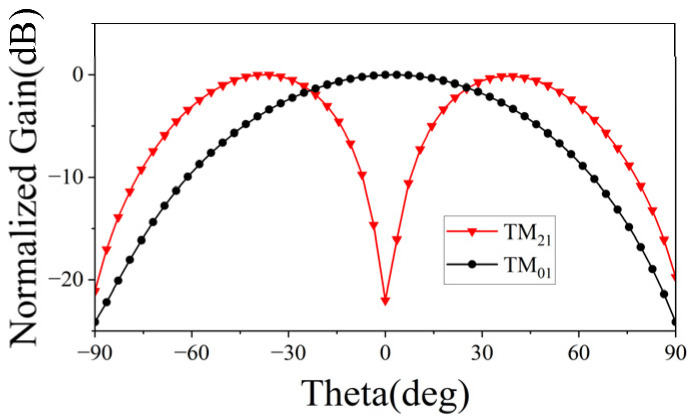
Far-field patterns of TM_01_ and TM_21_ modes under ideal conditions.

**Figure 3:**
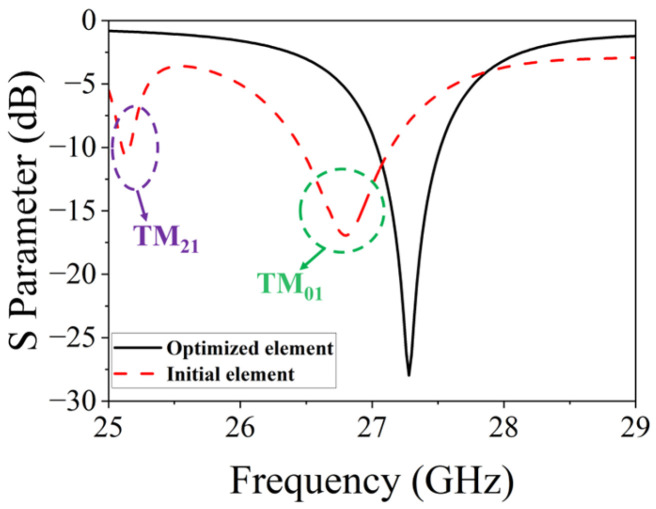
Frequency responses of the proposed element.

**Figure 4:**
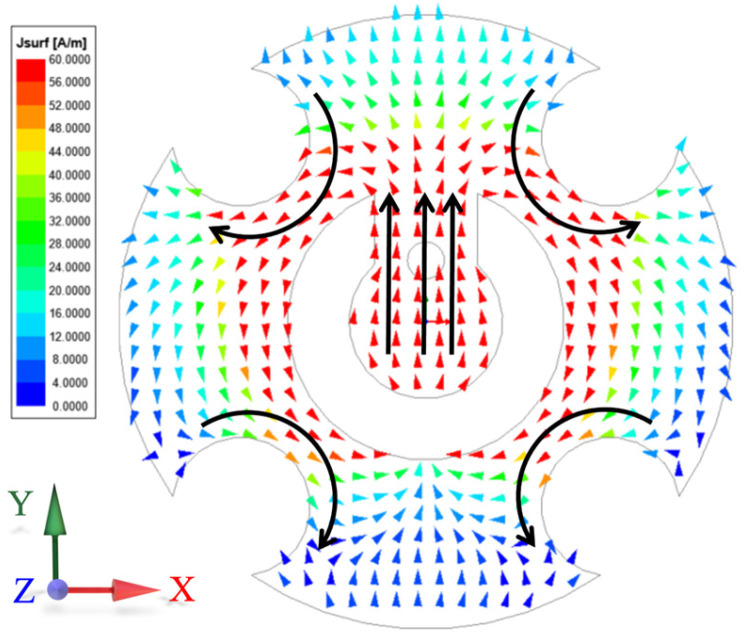
Surface current distribution of the slot-etched circular patch.

The element operation principle can be explained as follows. On one hand, the formation of the TM_01_ mode on the inner side of the split-ring slot can be achieved through the adjustment of the parameters *fx* and *Rs*. On the other hand, excitation of the TM_21_ mode on the exterior of the split-ring slot can be achieved by adjusting the parameters *Rd* and *dc* of the four diagonally located circular grooves. Figure 2 displays the far-field patterns of TM_01_ and TM_21_ modes under ideal conditions. If the resonant frequencies of these two modes can be kept consistent, it is possible to achieve wide beam coverage in the joint mode. The frequency response of the initial element, depicted by the red dashed line in Figure 3, reveals a noticeable deviation in the resonant frequencies of the TM_01_ and TM_21_ modes. In order to maintain the same resonant frequency, a process of parameter optimization is executed. Alongside the aforementioned parameters, the parameters *sw* and *gw* are also of paramount significance, since they control the coupling between the TM_01_ and TM_21_ modes and turn the resonant frequency. The frequency response of the element based on the optimized parameters is depicted by the black solid line in Figure 3, while the optimal parameters of the element are enumerated as follows: *Rp* = 2.3 mm, *L* = 5.7 mm, *w* = 0.3 mm, *Rs* = 0.9 mm, *gw* = 0.4 mm, *sw* = 0.6 mm, *Rd* = 0.5 mm, *dc* = 1.9 mm, *Rm* = 0.1 mm, *ds* = 0.6 mm, *hs* = 2.286 mm, *fx* = 0.4 mm, *Rv* = 0.12 mm, *w*1 = 1.43 mm, *w*2 = 0.5 mm, *w*3 = 0.73 mm, *L*1 = 0.95 mm, *L*2 = 2.26 mm, *L*3 = 1.16 mm. Based on these optimized parameters, the element exhibits a single resonance point at 27.3 GHz, which is also the same resonance frequency of the TM_01_ mode and TM_21_ mode. Hence, this study employs a center frequency of 27.3 GHz for the design of this wide-beamwidth element. The surface current distribution of the slot-etched circular patch depicted in Figure 4; it exhibits a distinct TM_01_ mode within the split-ring slot and a TM_21_ mode outside the split-ring slot, thereby validating the precision of the optimization procedure.

**Figure 5:**
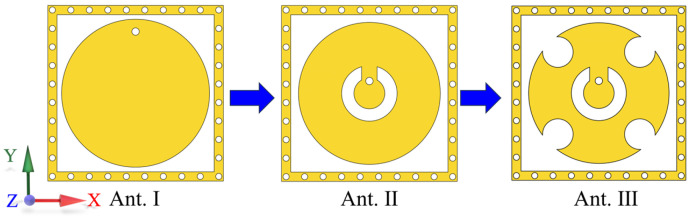
Evolutionary progression of the element.

**Figure 6:**
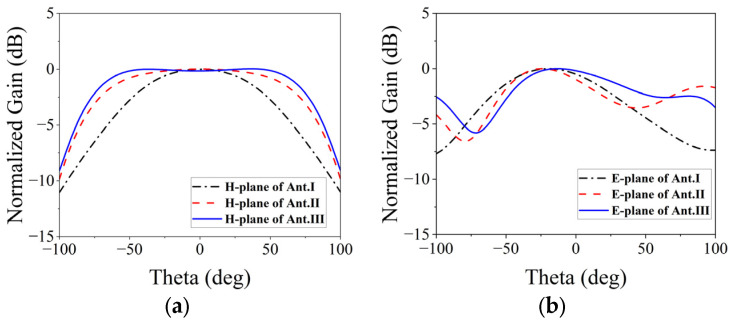
Normalized far-field patterns for the three elements: (**a**) H-plane; (**b**) E-plane.

To illustrate the benefits of the proposed element, Figure 5 and Figure 6 present the evolutionary progression of the element’s structure and the corresponding far-field patterns at both planes. The conventional circular patch is denoted as Ant. I, while an additional split-ring slot is designated as Ant. II. Ant. III, introduced in this study, features a circular patch with etched slots. To ensure equitable comparison, all three antennas employ dielectric substrates of identical dimensions and thicknesses, with impedance matching achieved through parameter adjustments. The simulated beamwidth and gain of the three element structures are presented in Table 1. Analysis of the data in this table reveals that Ant. I exhibit the narrowest beamwidth, resembling traditional patch antennas, resulting in a beamwidth of approximately 100°. However, this type of antenna demonstrates the highest gain due to the inverse relationship between gain and beamwidth. Conversely, Ant. III displays the lowest gain and widest beamwidth. This proposed structure is validated by the substantial improvement in beamwidth at both planes observed from Ant. I to Ant. III.

## 3. Linear Phased Arrays: Design and Analysis

**Figure 7:**
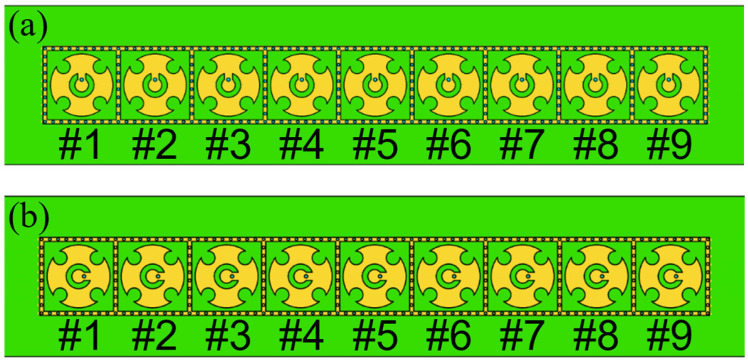
Two phased arrays: (**a**) H-plane; (**b**) E-plane.

**Figure 8:**
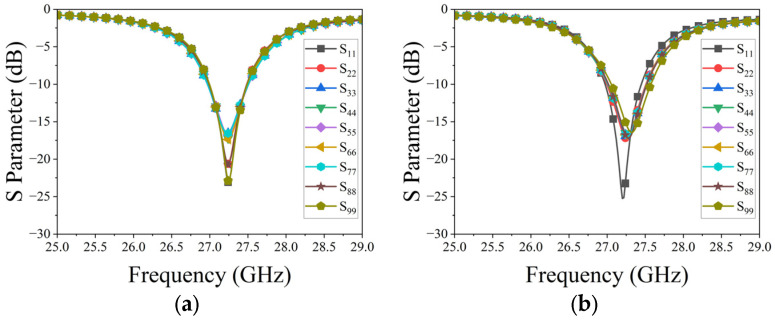
Reflection coefficients of the phased arrays: (**a**) H-plane; (**b**) E-plane.

**Figure 9:**
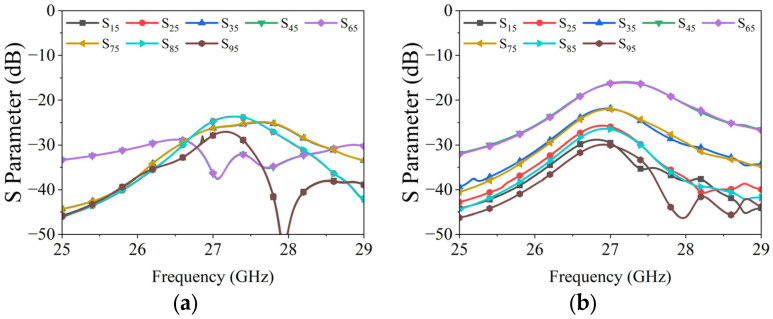
Mutual coupling of the phased arrays: (**a**) H-plane; (**b**) E-plane.

Figure 7 illustrates two linear phased arrays, each composed of nine uniformly connected elements. To avoid grating lobes in periodic linear phased arrays, an inter-element spacing of 5.4 mm was selected for both arrays, roughly equivalent to 0.4λ at 27.3 GHz. The dimensions of 75.78 mm × 11.7 mm for both arrays were chosen for ease of future installation and experimentation. Reflection coefficients for each element in the arrays, calculated using the CST Microwave studio, are shown in Figure 8. The CST Microwave studio employs 25 mesh lines per wavelength and a 50 dB accuracy level to ensure high simulation precision. The results indicate that at 27.3 GHz, the reflection coefficients for each element in both arrays remain below −15 dB. Figure 9 depicts the mutual coupling between the arrays, measuring below −15 dB in both planes due to optimal inter-element spacing and the arrangement of metallized holes in square rings.

**Figure 10:**
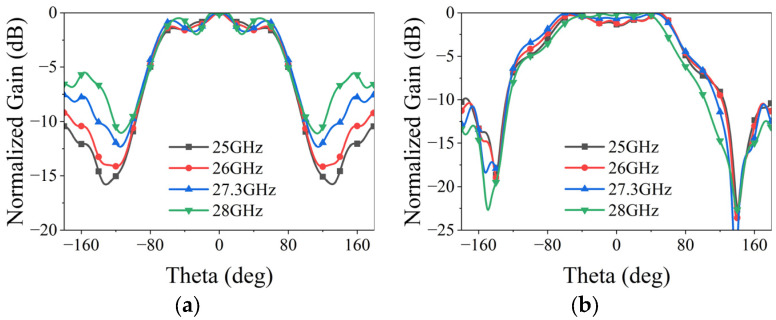
Simulated AEPs of the center element of the phased arrays: (**a**) H-plane; (**b**) E-plane.

The radiation patterns of the proposed single element are shown in Figure 6. To assess the actual scanning performance of the fully excited array, the active element pattern (AEP) is utilized. This pattern is obtained by exciting one specific element while terminating all other elements with matched loads, effectively accounting for mutual coupling effects [29]. Building on the analyses in [30,31], the AEP data allows extraction of the active input impedance for any element at various scanning angles, enabling the prediction of blind spots. Figure 10 illustrates the simulated AEPs of the central element (Element 5) on both planes. In contrast to the radiation pattern of the single element, the AEPs exhibit more fluctuations due to varying radiation environments and the influence of neighboring elements. Notably, at 27.3 GHz, the 3 dB beamwidth of the central element on both planes exceeds 140°, confirming the wide-angle radiation capability of the proposed element.

**Figure 11:**
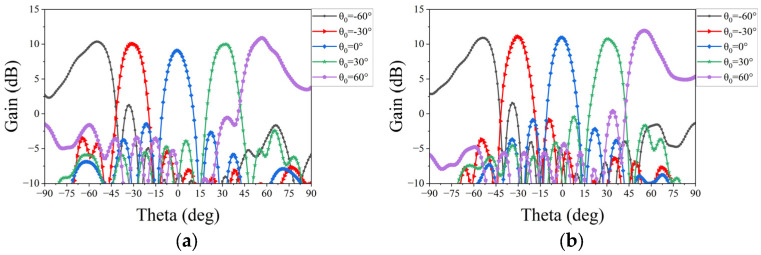

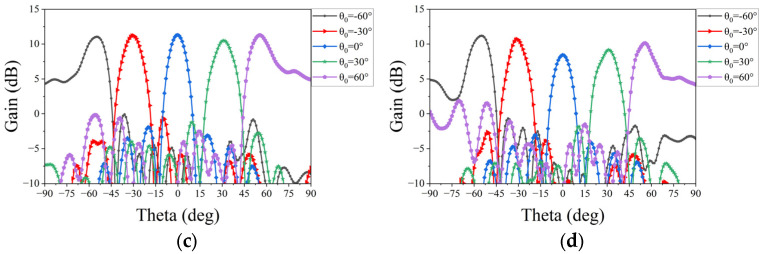
Simulated patterns of the proposed phased array at the E-plane: (**a**) at 25 GHz; (**b**) at 26 GHz; (**c**) at 27.3 GHz; and (**d**) at 28 GHz.

**Figure 12:**
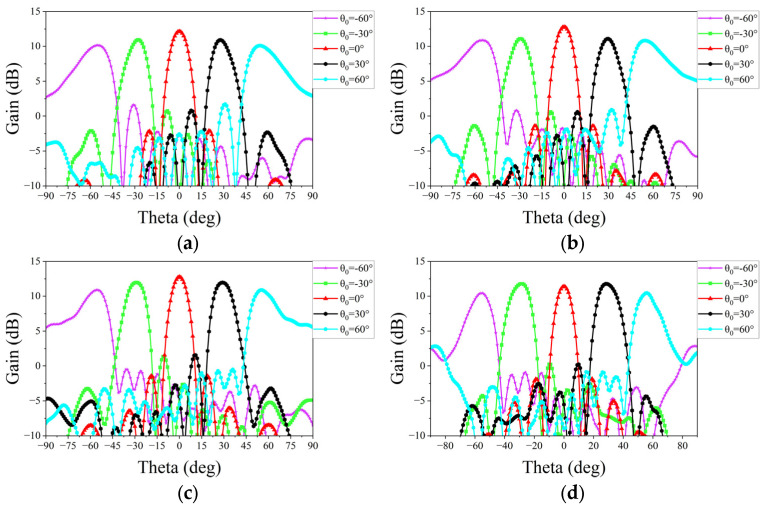
Simulated patterns of the proposed phased array at the H-plane: (**a**) at 25 GHz; (**b**) at 26 GHz; (**c**) at 27.3 GHz; and (**d**) at 28 GHz.

The simulated scanning performance of the entire array is directly obtained from CST Microwave studio by driving all of the feeding ports simultaneously. The realized gain patterns of two phased arrays are synthesized from all the simulated AEPs, which is calculated as follows:

(1) $$\left\{\begin{array}{l} S(\theta ,\varphi )=\sum_{i=1}^{N}a_{i}S_{iAEP}(\theta ,\varphi )e^{j[k_{0}(N-i)d(u+v)+\psi_{i}]}\\ u=\sin\theta \times \cos\varphi \\ v=\sin\theta \times \sin\varphi \end{array}\right.$$

where *d* is the inter-element spacing, *N* is the total number of the linear phased array (which is set to nine), *S_iAEP_* represents the AEP within the linear phased array, and *k*_0_ is the wave number. The coefficients *a_i_* collectively create an amplitude taper, with this study employing a uniform amplitude distribution. Additionally, $\psi_{i}$ denotes the input phase. Here, we choose a linear phase taper that is equal to:

(2) $$\left\{\begin{array}{l} \psi_{i}=-k_{0}(N-i)d(u1+v1)\\ u1=\sin\theta_{0}\times \cos\varphi_{0}\\ v1=\sin\theta_{0}\times \sin\varphi_{0} \end{array}\right.for\;i=1,2,\ldots ,N$$

where *θ*_0_ and *φ*_0_ represent the desired scanning angle of the linear phased array. The synthesized results are illustrated in Figure 11 and Figure 12. These show that the scanning beam of the two phased arrays can cover a range from −60° to 60° at 25–28 GHz with a gain loss of within 3 dB.

## 4. Measurement Results of Two Phased Arrays

**Figure 13:**
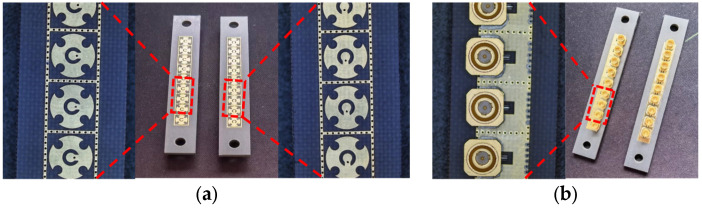
Prototypes of the arrays: (**a**) top view; (**b**) bottom view.

Finally, the two linear phased arrays, manufactured using PCB technology, share identical dimensions of 75.78 mm × 11.7 mm. These arrays feature SSMP connectors as their feeding ports, as depicted in Figure 13. To validate the actual performance of these arrays, two experiments were conducted.

**Figure 14:**
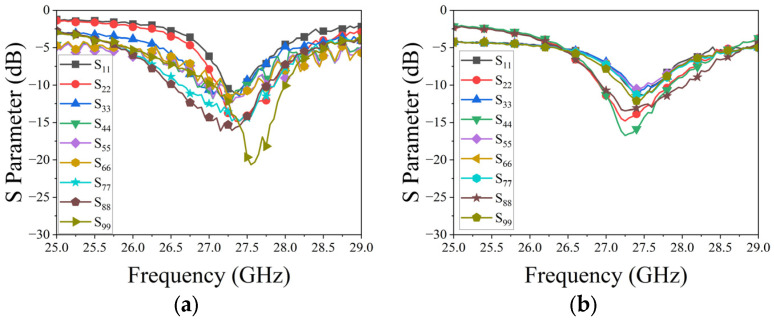
Measured reflection coefficients of each element in two arrays: (**a**) E-plane; (**b**) H-plane.

The reflection coefficients of each element in the two arrays are measured using the Ceyear 3672C vector network analyzer (VNA). Figure 14 displays the measured results for both linear phased arrays. A comparison with Figure 8 reveals a slight shift in the measured resonance frequency of the elements and an increase in the measured values. These deviations primarily stem from processing and assembly errors. However, despite these discrepancies, the overall results align with the design requirements for the linear phased array.

**Figure 15:**
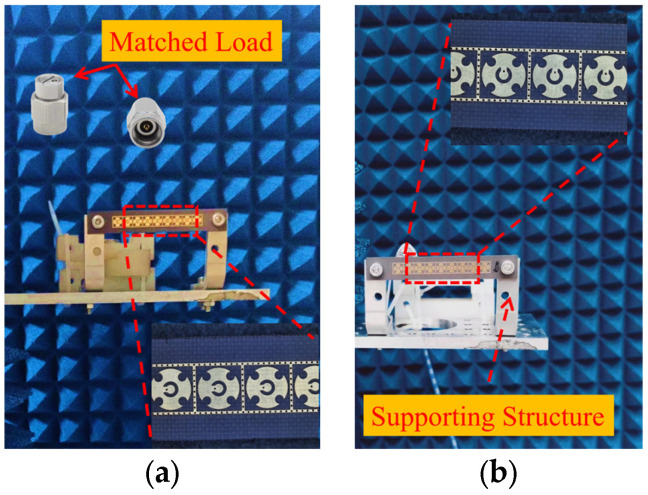
Measurement setup within an anechoic chamber: (**a**) E-plane array; (**b**) H-plane array.

**Figure 16:**
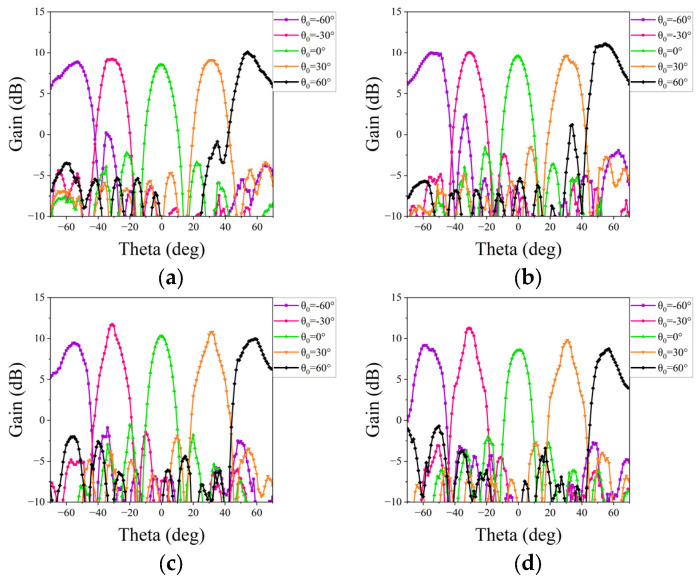
Measured patterns of the proposed phased array at the E-plane: (**a**) at 25 GHz; (**b**) at 26 GHz; (**c**) at 27.3 GHz; and (**d**) at 28 GHz.

**Figure 17:**
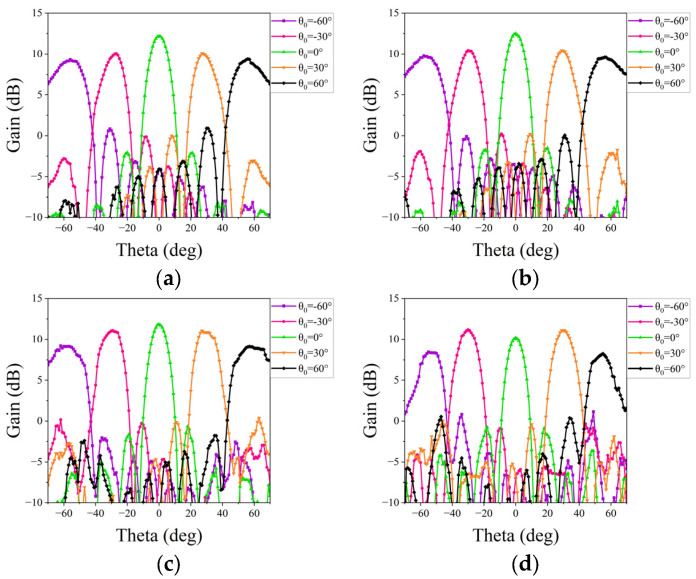
Measured patterns of the proposed phased array at the H-plane: (**a**) at 25 GHz; (**b**) at 26 GHz; (**c**) at 27.3 GHz; and (**d**) at 28 GHz.

Subsequently, a measurement is conducted to evaluate the beam scanning capability of two linear phased arrays. Equations (1) and (2) are then utilized to synthesize radiation patterns for each scanning angle, requiring the initial acquisition of the AEPs. Each AEP is obtained by exciting a single port of the array while terminating all other ports with matched loads. Based on this, nine AEPs for each array are measured individually in the microwave anechoic chamber by sequentially exciting each feeding port. The assembly diagram in Figure 15 illustrates the setup within the chamber, wherein the phased array is secured to the antenna turntable. Rotating the turntable allows us to obtain the AEPs for both main planes. Figure 16 and Figure 17 show the experimental results of the synthesized patterns for the two phased arrays. It is evident that, despite the presence of processing and testing errors leading to a slight decrease in beam gain at each frequency point com-pared to the simulation results, the beam remains capable of achieving scanning within a range of −60° to 60°. Moreover, the beam gain fluctuation remains under 3 dB, affirming the accuracy and effectiveness of the design.

## 5. Conclusions

This paper introduces a novel single-layer wide-beamwidth element that employs simultaneous excitation of the TM_01_ and TM_21_ modes on a slot-etched circular patch. This approach enables the achievement of a width by adjusting the parameters and the feeding position. To address mutual coupling among array elements, strategically positioned metallized holes surrounding the circular patch are utilized. Both the simulated and measured results demonstrate wide-angle scanning with minimal gain fluctuation in the arrays. This method could serve as a valuable reference for designing phased arrays with wide-angle scanning capabilities. Future research should aim to explore integrating characteristic mode analyses and other methodologies to further enhance phased array performance.

## Figures and Tables

**Table 1:** Comparison table of the gain and beamwidth for the three element structures.

	*HPBW in E-Plane*	*HPBW in H-Plane*	*Peak Gain (dBi)*
Ant. I	*98.3°*	*104°*	*4.32*
Ant. II	*84°*	*148.7°*	*3.36*
Ant. III	*146.7°*	*159°*	*2.85*

## Data Availability

The data supporting the findings of this study can be made available to genuine readers after contacting the corresponding authors. The data are not publicly available due to privacy.
